# Variability of reagents matters—enhancements to the CLSI modified carbapenem inactivation method outside the United States to improve accuracy

**DOI:** 10.1128/jcm.01509-24

**Published:** 2025-02-04

**Authors:** Sweety Singh, Shaoli Basu, Shahzad Mirza, Rajesh Karyakarte, Camilla Rodrigues, Yehudit Bergman, Mrunmayi Naik, Bharat Randive, Emily Jacobs, Anushruti Gupta, Matthew L. Robinson, Patricia J. Simner

**Affiliations:** 1 BJ Government Medical College-Johns Hopkins Clinical Research Site, Pune, India; 2 Johns Hopkins Center for Infectious Diseases in India, Pune, India; 3 Department of Laboratory Medicine, P. D. Hinduja National Hospital and Medical Research Centre 29537 , Mumbai, Maharashtra, India; 4 Department of Microbiology, Dr. D. Y. Patil Medical College, Hospital and Research Centre, Dr. D. Y. Patil Vidyapeeth University 75141 , Pune, Maharashtra, India; 5 Department of Microbiology, Byramjee Jeejeebhoy Government Medical College, Pune, India; 6 Department of Pathology, Johns Hopkins University School of Medicine 1500 , Baltimore, Maryland, USA; 7 Johns Hopkins Center for Infectious Diseases in India, Baltimore, Maryland, USA; 8 Department of Medicine, Johns Hopkins University School of Medicine 1466 , Baltimore, Maryland, USA; Marquette University, Milwaukee, Wisconsin, USA

**Keywords:** carbapenem-resistant organisms, carbapenemase, modified carbapenem inactivation method

## LETTER

Carbapenem-resistant gram-negative organisms (CRO) are broadly divided by mechanism mediating resistance into (i) carbapenemase-producing (CP)-CRO and (ii) non-carbapenemase-producing (non-CP) CRO. CP-CRO is more aggressively targeted by infection prevention and control (IPC) and antimicrobial stewardship programs, as it is the predominant mechanism driving carbapenem resistance globally, and infections are more challenging to treat (1). Thus, the detection of CP-CRO by clinical microbiology laboratories should be performed to support patient treatment decisions and for IPC and public health initiatives (2).

The Clinical Laboratory Standard Institute (CLSI) introduced the modified carbapenem inactivation method (mCIM) as a method for detecting and distinguishing CP-CRO from non-CP-CRO. This method utilizes readily available supplies in laboratories with a potential global reach. At the time of endorsement, the assay demonstrated good sensitivity (>97%) and specificity (>95%) for the detection of CP-*Enterobacterales* and *Pseudomonas aeruginosa* in two US multicenter studies across various media and disk manufacturers (3, 4).

We evaluated the CLSI mCIM at three different hospitals within India (P.D. Hinduja Hospital and Medical Research Centre, Mumbai; BJGMC and Sassoon General Hospital, Pune and Dr DY Patil Medical College, Hospital and Research Centre, Pune) to detect CP- *Enterobacterales* and *P. aeruginosa*. Upon local validation of the method, discrepant results for the detection of Class B & D enzymes were encountered. Based on previous reports demonstrating the importance of cation content for detection of Class B (i.e., requirement of zinc at the active site for metallo-beta-lactamase [MBL] activity) and D (i.e., more active dimeric form in the presence of cations) enzymes and the necessity of a higher inoculum for carbapenemases with weak enzymatic activity (e.g., OXA-48-like), we evaluated modifications to the mCIM to identify CP-CRO among ertapenem and/or meropenem not susceptible (e.g., intermediate or resistant) isolates based on standard of care methods as each site (Vitek 2AST-N405, -N406, -N407, bioMerieux at sites 1 and 3 or disk diffusion at site 2), as appropriate for the organism (Table S1). The modifications included the use of cation-adjusted Mueller Hinton broth (CAMHB) in place of tryptic soy broth (TSB) and an increased inoculum for the Enterobacterales from 1 µL to 10 µL (5, 6). We hypothesized that the cation content of the broth and increased inoculum would lead to improved detection of Class B and D enzymes. Thus, a set of 29 previously phenotypically and whole genome sequenced (WGS) CRO isolates were evaluated, including 12 non-CP-CRO and 17 CP-CRO (5 KPC, 4 OXA-48-like, 2 NDM, 3 NDM & OXA-48-like, 2 VIM, and 1 SME; Table S2). The mCIM was performed following four different protocols to attempt to improve the accuracy of detection of Class B and D CP-CRO while maintaining specificity: (i) CLSI: The CLSI mCIM procedure with TSB (HiMedia; Mumbai, India) using a 1 µL and 10 µL inoculum for Enterobacterales and *P. aeruginosa*, respectively (2); (ii) TSB 10: mCIM with TSB and 10 µL inoculum for all organisms; (iii) CAMHB: mCIM with CAMHB (Millipore [Wilmington, DE] BJGMC; HiMedia: Hinduja and DY Patil) and 1 µL inoculum for all organisms; and (iv) CAMHB 10: mCIM with CAMHB and 10 µL inoculum for all organisms. Biorad [Boston, MA] meropenem disks were used after quality control demonstrated a larger-than-expected zone for HiMedia meropenem disks with *Escherichia coli* ATCC 25922 (7).

On analysis, we found that the overall accuracy of the mCIM methods ranged from 84% with the CLSI method to 99% using CAMHB 10 method (Table 1). The sensitivity when compared to WGS was 73% following the CLSI method to 98% using CAMHB 10. Discrepant results are summarized in Table 1 and most commonly occurred with Class B and D producers, including dual producers. The specificity was 100% for all methods. The reproducibility of the methods across sites ranged from 84% for the mCIM CLSI, to 93% for TSB 10, to 98% for CAMHB, and 99% for CAMHB 10. The mCIM using CAMHB with 10 µL inoculum for all CRO improved the accuracy and reproducibility of detecting CP-CRO, especially Class B and D enzymes. The method has since been implemented at all testing sites with BioRad meropenem disks. A follow-up retrospective assessment of the CAMHB 10 method demonstrated a 100% sensitivity (56/56 positives) and 95% specificity (19/20 negatives) when compared with local WGS of 76 isolates (Table S1). A false-positive mCIM was encountered for *P. aeruginosa* when compared with WGS results.

**TABLE 1:** Summary of mCIM results across three sites in India

Site	mCIM method	No. of isolates	No. of discrepant results	Accuracy	Sensitivity	Specificity
Site 1	CLSI	29	7^*a*^	76%	59%	100%
	CAMHB 10	29	0	100%	100%	100%
Site 2	CLSI	29	4^*b*^	86%	76%	100%
	TSB 10	29	3^*c*^	90%	82%	100%
	CAMHB	29	2^*d*^	93%	88%	100%
	CAMHB 10	29	1^*e*^	97%	94%	100%
Site 3	CLSI	29	3^*f*^	90%	82%	100%
	TSB 10	29	1^*e*^	97%	94%	100%
	CAMHB	29	1^*g*^	96%	93%	100%
	CAMHB 10	29	0	100%	100%	100%

^*a*^ Discrepant results include VIM-2-producing *P. aeruginosa*, OXA-181-producing *Escherichia coli* (2), OXA-232 *Klebsiella pneumoniae*, NDM-1 and OXA-232-prouducing *K. pneumoniae*, OXA-48 producing *E. coli,* NDM-1 and OXA-181-prouducing *Providencia rettgeri*, NDM-7 and OXA-181 producing *E. coli.*

^*b*^ Discrepant results include VIM-2-producing *P. aeruginosa* , inconclusive OXA-48-producing *E. coli,* inconclusive NDM-1 and OXA-232-prouducing *K. pneumoniae*, and NDM-1 and OXA-181-prouducing *Providencia rettgeri.*

^*c*^ Discrepant results include VIM-2 producing *P. aeruginosa* OXA-181-like-producing *E. coli,* and NDM-1 and OXA-232-prouducing *K. pneumoniae.*

^*d*^ Discrepant results include VIM-2 producing *P. aeruginosa* and NDM-1 and OXA-181-prouducing *P. rettgeri.*

^*e*^ Discrepant result was a VIM-2 producing *P. aeruginosa*.

^*f*^ Discrepant results include, inconclusive OXA-181-producin*g E. coli*, NDM-1 and OXA-232 prouducing *K. pneumoniae*, NDM-1 and OXA-181-prouducing *P. rettgeri.*

^*g*^ Discrepant result was a NDM-1 and OXA-181-prouducing *P. rettgeri*.

This study demonstrates the importance of QC and validation of the mCIM method in different geographic areas outside the US to ensure performance as the epidemiology of CP-CRO varies and due to differences in availability and quality of supplies required for testing (e.g., TSB and meropenem disks).

## SUPPLEMENTAL MATERIAL

Supplemental tables jcm.01509-24-s0001.xlsx Tables S1 and S2.

ASM does not own the copyrights to Supplemental Material that may be linked to, or accessed through, an article. The authors have granted ASM a non-exclusive, world-wide license to publish the Supplemental Material files. Please contact the corresponding author directly for reuse.
